# McLaughlin technique and humeral grafting provide similar results for treatment of reverse Hill–Sachs lesions: A systematic review

**DOI:** 10.1002/jeo2.12001

**Published:** 2024-03-10

**Authors:** Matteo Buda, Vito Coco, Alberto Grassi, Mattia Filanti, Costanza Musiani, Luca Solaro, Enrico Guerra, Matteo Romagnoli

**Affiliations:** ^1^ Ortopedia e Traumatologia Rizzoli Argenta Argenta Ferrara Italy; ^2^ II Clinica Ortopedica e Traumatologica IRCCS Istituto Ortopedico Rizzoli Bologna Italy; ^3^ Chirurgia della Spalla e del Gomito IRCCS Istituto Ortopedico Rizzoli Bologna Italy

**Keywords:** anterior shoulder depression, McLaughlin, posterior shoulder dislocation, reverse Hill–Sachs, shoulder instability

## Abstract

**Purpose:**

Various surgical treatments have been described for the treatment of reverse Hill–Sachs lesions (rHSls) sized between 20% and 50% in the case of posterior shoulder dislocation. The aim of this systematic review is to report the clinical and radiological outcomes of subscapularis or lesser tuberosity transfer (McLaughlin and modified procedures) compared to bone or osteochondral autograft or allograft.

**Methods:**

A systematic review was performed on five medical databases up to December 2022. The inclusion criteria were clinical studies of all levels of evidence describing clinical or radiological outcomes of either procedure. The assessment of the quality of evidence was performed with the Modified Coleman Score.

**Results:**

A total of 14 studies (five prospective and nine retrospective) were included. A total of 153 patients (155 shoulders, 78.4% male) with a mean age of 37.2 (22–79) years were reviewed at an average follow‐up of 53.1 (7.1–294) months. No relevant difference was found for the clinical scores, range of motion, complications and redislocation rate between the two treatments. Radiological osteoarthritis (OA) was reported in 11% (10/87) in the McLaughlin group and in 21% (16/73) in the humeral reconstruction group.

**Conclusions:**

McLaughlin and anatomic humeral reconstruction lead to similar satisfactory clinical results and a low redislocation rate in the treatment of rHSls. Anatomic humeral reconstruction seems associated with an increased risk of OA progression.

**Level of Evidence:**

Level IV.

**Abbreviations**
ABD: abduction
ADL: activity of daily living
ASES: American Shoulder and Elbow Surgeons Shoulder Score
CS: Constant‐Murlay Score
ER: external rotation
F: female
FF: forward flexion
FH: femoral head
HH: humeral head
IC: iliac crest
IR: internal rotation
M: male
mCMS: Modified Coleman Methodology Score
ML: McLaughlin
n/a: not available
OA: osteoarthritis
P: prospective
PRISMA: Preferred Reporting Items for Systematic Reviews and Meta‐Analyses
R: retrospective
rHSls: reverse Hill‐Sachs lesions
SST: Simple Shoulder Test
SSV: subjective shoulder value
UCLA: University of California, Los Angeles Shoulder Score
VAS: Visual Analogue Scale
WOSI: Western Ontario Shoulder Instability Index

## INTRODUCTION

Posterior shoulder dislocation is relatively rare, accounting for only 1%–5% of all shoulder dislocations [1]. Possible causes of posterior glenohumeral dislocations include epileptic seizures, electric shocks, direct or indirect trauma with shoulder flexion, adduction, and internal rotation or anteroposterior trauma with abducted and externally rotated shoulder [1]. Despite the typical mechanisms of injury, clinical presentation and radiological signs, the diagnosis is often missed during initial assessment [2].

A pathognomonic humeral head anterior impression fracture, known as the McLaughlin lesion or reverse Hill–Sachs lesion (rHSl), is frequently present, particularly in cases of chronic posterior locked shoulder dislocation [3]. This lesion can cause significant clinical symptoms, early joint damage, and osteoarthritis (OA). Moreover, depending on the size and location, rHSls are an important risk factor for redislocation as they engage with the posterior aspect of the glenoid rim and often require surgical treatment [3].

The humeral bone defect can be quantified and measured on axial CT scans, between the posterior defect edge and the bicipital groove [4]. While small defects (<25% of articular cartilage) are treated conservatively with open or closed reduction, and large defects (>50%) are treated with shoulder replacement [1], the treatment of medium‐sized defects (25%–50%) remains controversial [5].

Various surgical treatments have been described for the management of medium impression fractures, such as medial transposition of the lesser tuberosity [6], defect‐filling with autograft or allograft [7, 8], posterior bone block [9], derotational osteotomy [10], retrograde elevation of the impression fracture [11] and arthroscopic or open remplissage with subscapularis tendon [12, 13].

Currently, the two most commonly used surgical procedures for humeral anterior bone defects are the McLaughlin technique and the autograft or allograft filling of the defect [14]. In 1952, McLaughlin described the procedure in which the subscapularis tendon was detached from its origin and subsequently inserted into the defect [6]. In 1987, Hawkins et al. modified the technique by performing an osseous transfer of the lesser tuberosity and its attached subscapularis into the defect [15]. Over the years, several authors have revisited the McLaughlin procedure, describing variations to the original technique [16]. Anatomical humeral reconstruction with osteochondral allograft was first published by Gerber et al. in 1996 [17]. Subsequently, different case series and new surgical techniques were described using various grafts, autologous or from tissue banks [14].

The purpose of this systematic review was to evaluate clinical and radiographic outcomes, complications (including OA, screw impingement, loosening, breakage and graft resorption) and loss of stability in patients undergoing surgery to restore rHSl by comparing the McLaughlin technique with graft filling. Our null hypothesis was that no superiority of any technique would be found based on the available literature.

## MATERIALS AND METHODS

### Search strategy and article selection

**Figure 1:**
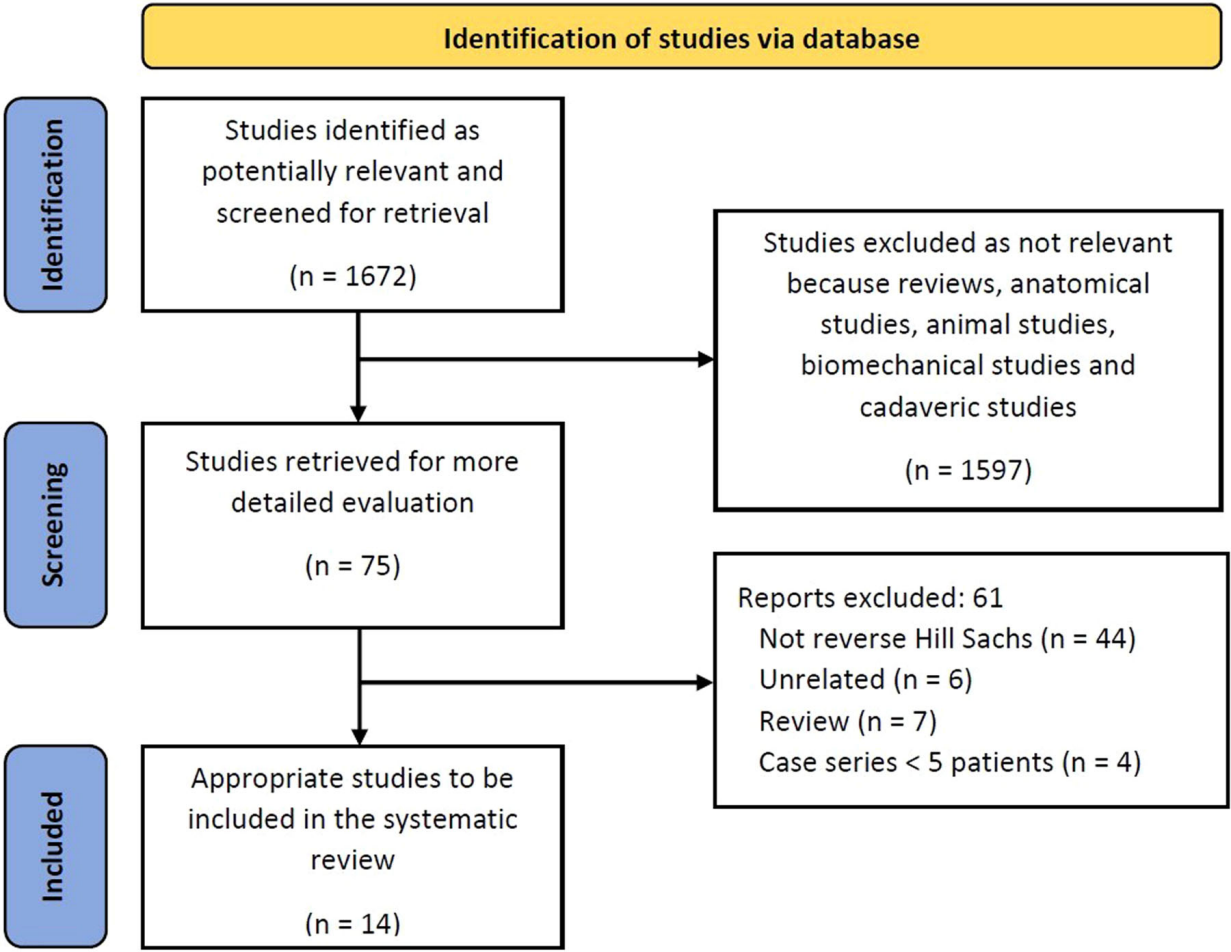
Preferred Reporting Items for Systematic Reviews and Meta‐Analyses flowchart.

Following the Oxford Center of Evidence‐Based Medicine guidelines, we conducted a systematic review of all Level I–IV studies published in English on three electronic databases (PubMed, Cochrane and Web of Science) from January 1990 to December 2022 that described the treatment and outcomes of posterior dislocation with rHSls using either the McLaughlin technique or osteochondral graft filling [18]. The study adhered to the Preferred Reporting Items for Systematic Reviews and Meta‐Analyses (PRISMA) guidelines for article identification [19] (Figure 1).

The review was registered on the PROSPERO database (CRD42022375789). The following research terms were used: ‘(posterior shoulder dislocation) AND (reverse Hill–Sachs) AND (McLaughlin OR anterior shoulder depression OR shoulder instability)’.

Articles reporting clinical and/or radiological outcome data in patients who underwent surgical treatment for the rHSl procedure were included. Case reports or case series describing less than five cases and articles in languages other than English were excluded. Preclinical, ex vivo studies, congress abstracts and review articles were also excluded. Reference lists from the selected papers and from the systematic reviews found with the first and second screening were also considered, and all selected studies were included in the qualitative data synthesis.

### Data extraction, assessment of risk of bias and quality of evidence

The articles were analyzed based on their title and abstract by two independent investigators (M. B. and V. C.). In case of disagreement, a third investigator (L. S.) was consulted to reach a consensus.

The studies were assessed for methodological research quality using the Modified Coleman Methodology Score (mCMS) criteria [20]. Each study was evaluated, and a total score ranging from 0 to 100 points was assigned. The final score was categorized as excellent (85–100 points), good (70–84 points), fair (50–69 points) or poor (<50 points).

The reviewers extracted independently relevant data from the included articles, such as the year of publication, study type, number of patients, demographic data, diagnosis, type of surgical procedure, follow‐up duration, outcomes and complications. The extracted data were discussed to minimize selection bias and errors, and they were summarized in tables using Microsoft Excel (2013 version; Microsoft Corporation). No contact was made with the authors to verify the accuracy of the data or obtain further information.

## RESULTS

### Literature review

A total of 1772 relevant publications were initially identified through electronic search. After applying the inclusion criteria, 75 studies remained. Out of these, 61 studies were excluded because they were case reports, technical notes or did not meet the inclusion criteria. Ultimately, 14 studies were included in the review, consisting of nine retrospective [7, 8, 21, 22, 23, 24, 25, 26, 27] and five were prospective [28, 29, 30, 31, 32]. A summary of the study selection process is reported in the PRISMA diagram (Figure 1). Regarding the investigated procedures, seven studies reported the results of McLaughlin procedures and seven reported the results of humeral reconstruction by defect filling with femoral head [25, 31] or humeral head [8, 26] allograft or iliac crest autograft [28] or both [7, 27]. The evaluation with the mCMS showed an overall poor quality of the included articles with a mean of 67.3 (range 57–82). No improvement was found over time for the mCMS score of the published articles. Results are reported in Table 1.

**Table 1:** Methodological quality of the included studies with Coleman score.

References	Part A	Part B	A + B
Castagna et al. [22]	40	28	68
Banerjee et al. [21]	34	31	65
Kokkalis et al. [30]	44	28	72
Shams et al. [32]	44	29	73
Demirel et al. [24]	34	23	57
Khira and Salama [29]	44	31	75
Cohen et al. [23]	34	28	62
Bock et al. [28]	50	32	82
Diklic et al. [25]	37	28	65
Gerber et al. [7]	30	28	58
Murphy et al. [31]	44	28	72
Mi et al. [27]	34	28	62
Marcheggiani et al. [8]	37	31	68
Martinez et al. [26]	40	23	63

### Demographics

The analysis included a total of 155 shoulders in 153 patients, 140 were male. The mean age of the patients was 42 years, ranging from 22 to 79 years (37 years in the McLaughlin group compared to 46.4 years in the defect‐filling group). The McLaughlin procedure was performed in 80 patients (81 shoulders), while humeral reconstruction with auto or allograft was performed in 73 patients (74 shoulders). The mean follow‐up duration was 54.4 months, with a longer follow‐up period observed in the defect‐filling group (69.9 months) compared to the McLaughlin group (38.9 months). In all studies analyzed, the surgical treatment was performed in humeral head defects ranging between 20% and 50%. A postoperative protocol involving temporary immobilization of the shoulder in a sling for 4–6 weeks, followed by a rehabilitation program. Demographic data are reported in Table 2.

**Table 2:** A summary of the demographic data from the various publications.

References	Study design	No. (M/F)	Shoulders	Age (y)	Defect	Technique	Follow‐up (mo)
Castagna et al. [22]	R	16 (16)	16	41.9	20%–50%	ML	n/a
Banerjee et al. [21]	R	13 (12/1)	13	39	32% (±6.4)	ML	41
Kokkalis et al. [30]	P	5 (6, 4/1)	6	n/a	38% (30–45)	ML	20
Shams et al. [32]	P	11 (9/2)	11	39	35% (30–40)	ML	29
Demirel et al. [24]	R	13 (13)	13	39.3	27% (20–40)	ML	30
Khira and Salama [29]	P	12 (10/2)	12	26	40% (30–45)	ML	30
Cohen et al. [23]	R	10 (9/1)	10	36.3	32% (22–35)	ML	59.4
Bock et al. [28]	P	6 (5/1)	6	52.5	35.8 (30–45)	IC autograft	62.7
Diklic et al. [25]	R	13 (10/3)	13	42	25%–50%	FH allograft	54
Martinez et al. [26]	R	6 (6/0)	6	33	40%	HH allograft	122
Gerber et al. [7]	R	21 (22, 17/4)	22	44	43% (30%–55%)	17 FH or HH allograft 4 IC autograft	128
Murphy et al. [31]	P	5 (3/2)	5	53.4	30%–50%	FH allograft	34
Mi et al. [27]	R	10 (9/1)	10	44.8	33.95% (19.1–42.6)	Acute lesions: IC autograft Chronic lesions: HH or FH allograft	22.6
Marcheggiani et al. [8]	R	12	12	54.8	31% ± 1.32 (30–50)	HH allograft	66

Abbreviations: F, female; FH, femoral head; HH, humeral head; IC, iliac crest; M, male; ML, McLaughlin; n/a, not available; P, prospective; R, retrospective.

### Clinical outcomes

Various clinical scoring systems were used to assess postoperative outcomes, including the Constant and Murley Score (CS) [7, 8, 21, 22, 23, 24, 25, 26, 27, 28, 30, 31], the Western Ontario Shoulder Instability Index Score [8], the American Shoulder and Elbow Surgeons Shoulder Score [8, 21, 24], the Subjective Shoulder Value [7], the Simple Shoulder Test Score [22], the University of California, Los Angeles Shoulder Score [23, 29], the Visual Analogue Scale (VAS) for pain [23, 27] and range of motion evaluation [8, 21, 22, 24, 25, 26, 27, 28, 29, 30, 32].

Postoperative clinical scores increased in all evaluated studies. Although clinical scores differed considerably within each study, CS was the most commonly reported outcome measure. The mean postoperative CS was 80.2 for the McLaughlin group for 58 patients [21, 22, 23, 24, 30] and 82.7 for the defect‐filling group for 62 patients [7, 25, 26, 27, 28, 31], indicating no relevant difference. Details of the included articles are provided in Table 3.

**Table 3:** A summary of the mean clinical outcomes from the various publications.

References	CS	WOSI	ASES	SSV	SST	UCLA	VAS	ADL	FF	ABD	ER	IR
Castagna et al. [22]	75.2 (65–82)	n/a	n/a	n/a	1.2 (0–6)	n/a	n/a	n/a	151.8 (140–170)	138.1 (130–150)	51.8 (40–60)	41.8 (30–50)
Banerjee et al. [21]	92	n/a	98	n/a	n/a	n/a	n/a	n/a	175.7 (SD 4.9)	171.4 ± 6.4	54.3 ± 17.6	Waist—12th thoracic vertebra
Kokkalis et al. [30]	84 (77–90)	n/a	n/a	n/a	n/a	n/a	n/a	n/a	163 (150–175)	142 (130–155)	64 (50–80)	47
Shams et al. [32]	n/a	n/a	n/a	n/a	n/a	30 (20–34)	n/a	n/a	n/a	130 (110–155)	70 (55–80)	45 (35–55)
Demirel et al. [24]	85	n/a	78	n/a	n/a	n/a	n/a	n/a	163	151	70	n/a
Khira and Salama [29]	n/a	n/a	n/a	n/a	n/a	30 (28–33)	n/a	n/a	165 (150–175)	150 (145–160)	75 (60–80)	50 (40–60)
Cohen et al. [23]	65 ± 21.5	n/a	n/a	n/a	n/a	9.8 ± 1.3 (8–12)	2.4 ± 2.3	n/a	n/a	n/a	n/a	n/a
Bock et al. [28]	88.2 (83–98)	n/a	n/a	n/a	n/a	n/a	n/a	n/a	158.3 (150–160)	146.7 (140–160)	58.3 (50–60)	Lumbar spine segment III– thoracal VII
Diklic et al. [25]	86.8 (43–98)	n/a	n/a	n/a	n/a	n/a	n/a	17.2 (6–20)	155	n/a	n/a	n/a
Martinez et al. [26]	77 (52–98)	n/a	n/a	n/a	n/a	n/a	n/a	n/a	117.5 ± 40.2	n/a	69.2 ± 17.9	69.2 ± 17.9
Gerber et al. [7]	83 (45–96)	n/a	n/a	88 (60–100)	n/a	n/a	n/a	n/a	n/a	n/a	n/a	n/a
Murphy et al. [31]	91.7 ± 8.3	n/a	n/a	n/a	n/a	n/a	n/a	n/a	n/a	n/a	n/a	n/a
Mi et al. [27]	82 ± 15.09 (40–97)	n/a	n/a	n/a	n/a	n/a	0.68 ± 0.21	n/a	162.48 ± 12.68	n/a	n/a	n/a
Marcheggiani et al. [8]	n/a	11.20 ± 10.85	94 ± 8.49	n/a	n/a	n/a	n/a	n/a	166.6 ± 22.76	156.25 ± 25.09	82 ± 6.61	n/a

Abbreviations: ABD, abduction; ADL, activity of daily living; ASES, American Shoulder and Elbow Surgeons Shoulder Score; CS, Constant–Murlay Score; ER, external rotation; FF, forward flexion; IR, internal rotation; n/a, not available; SST, Simple Shoulder Test; SSV, subjective shoulder value; UCLA, University of California, Los Angeles Shoulder Score; VAS, Visual Analogue Scale; WOSI, Western Ontario Shoulder Instability Index.

### Postoperative imaging evaluation

Radiographic evaluation was carried out in four studies in the group of McLaughlin technique [21, 22, 23, 24] and in six articles of the defect‐filling group [7, 8, 25, 26, 28, 31]. The presence of radiographic shoulder OA varied among the studies. In the McLaughlin group, the cumulative rate of OA was 11% at a mean follow‐up of 30 months. In the defect‐filling group, 16 patients were diagnosed with osteoarthritic changes, resulting in an overall prevalence of 21.9% at a mean follow‐up of 80.8 months. Three cases of osteochondral allograft collapse and secondary osteonecrosis were also reported [25, 26]. Most of the studies did not specify the used OA classification: in two studies were adopted the Samuelson–Prieto classification [8, 23], while in one study was used the Kellgren Lawrence system [24].

### Complications, recurrence and reoperations

Different postoperative adverse events were reported in the included studies. One case of screw migration [21] and joint stiffness in two patients [29] were reported in the McLauglin group. Persistent pain at 3 months in one patient, one case of redislocation and one case of OA [28], posterior subluxation in one patient evidenced by CT scan [27], posterior subluxation and graft collapse in one patient [7] were reported in the humeral reconstruction group.

No reoperations were reported for the McLaughlin articles. Reoperations were performed in four patients from the humeral reconstruction group including reverse total shoulder arthroplasty for redislocation, posterior subluxation [28] and shoulder OA [7].

## DISCUSSION

The main finding of the present review is that both the McLaughlin procedure and defect filling demonstrate satisfactory outcomes for the treatment of posterior shoulder dislocation with rHSls sized between 20% and 50%.

The significance of humeral anteromedial impression fractures was initially recognized by McLaughlin, who described the subscapularis transfer as a surgical solution [6]. The McLaughlin technique was later modified by Hawkins et al., who performed an osteotomy transfer of the lesser tuberosity with the attached subscapularis tendon [15]. Currently, this technique remains one of the most frequently performed procedures due to its safety, reliability and cost‐effectiveness with excellent functional results and patient satisfaction [30].

Furthermore, it appears to be the most commonly used technique for small to medium‐sized defects, with better results when used in the acute setting [14].

Concerns regarding nonanatomic techniques, such as the McLaughlin procedure and its variations, are mainly related to the risk of subscapularis dysfunction, loss of strength or internal rotation and potential progression of OA [30]. In such cases, secondary joint replacement procedures would be technically more challenging and likely to yield lower clinical and functional outcomes [14].

Guehering et al. describes several surgical techniques for the treatment of the rHSl after posterior shoulder dislocation. In particular they reported a case series of five patients treated with retrograde elevation using arthroscopic assistance and a target device from knee ligament surgery, four patients treated with open reduction and antegrade corticocancellous cylindrical grafts and three patients with corticocancellous graft of the iliac crest. In conclusion, they observed best outcomes by using an autologous iliac crest corticocancellous bone graft, due to the lower secondary sintering rate of corticocancellous bone graft compared to retrograde elevation of the articular surface or antegrade cylindrical osteochondral grafting [33].

Anatomical humeral reconstruction with segmental osteochondral allograft using the femoral head to restore humeral articular shape was originally described in a case series by Gerber and Lambert [17]. Results have been satisfactory, and this technique is currently performed for medium‐sized defects with the aim of restoring normal osteoarticular anatomy and, therefore, enabling better shoulder function. However, graft collapse and head flattening can occur with this procedure. Diklic et al. [25] reported graft incorporation in 12 out of 13 patients, with one case of graft collapse and avascular necrosis, but satisfactory functional outcomes and no cases of recurrence. Humeral reconstruction with osteochondral allograft is usually preferred for medium to large defects (40%–50%) [14].

No difference was reported between the acute and chronic settings when performing humeral reconstruction with autograft or allograft defect filling.

Based on the findings of the present review, clinical outcomes, as evidenced by the Constant score, can be considered satisfactory for both the McLaughlin procedure and anatomical humeral graft defect filling. However, the choice of technique should consider the surgeon's confidence, patient‐specific factors, and external factors such as allograft availability and defect size.

Although no statistical analysis has been performed due to the limited and heterogeneous data, the radiological prevalence of OA appeared higher in the defect‐filling group. Moreover, the McLaughlin procedure is less invasive and seems associated with lower risk of complications/reintervention. However, it may not be as effective for larger lesions or cases involving damage to the subscapularis muscle. Nevertheless, an important data to take into consideration is a significantly longer follow‐up in the defect‐filling group compared to the McLaughlin group.

Lastly, longer follow‐ups and possibly higher‐level studies will be necessary before clear and evidence‐based guidelines can be established in the literature.

### Limitations

The present review has several limitations that should be acknowledged. Firstly, the included studies exhibited low quality and quantity, indicating a lack of robust literature in the field. This limits the strength of the conclusions drawn from the review. Citation mapping and grey literature searches were not performed in the present review. Additionally, most of the studies did not provide specific information regarding OA progression, subscapularis healing, and range of movement. These factors are crucial in determining the true superiority of one surgical technique over another. The absence of such data prevents a comprehensive evaluation of the outcomes.

## CONCLUSIONS

This systematic review showed that both the McLaughlin and anatomic humeral reconstruction lead to similar satisfactory clinical results and a low redislocation rate. It is important to note the lower risk of OA associated with the McLaughlin technique. Further studies with higher‐level designs are needed to confirm the findings of the present review and identify specific scenarios where one technique should be preferred over the other for the treatment of medium‐sized rHSls.

## AUTHOR CONTRIBUTIONS

All authors contributed equally to researching the data, analyzing articles, writing the paper and reviewing the final version of the manuscript. All authors read and approved the final manuscript.

## FUNDING

Funding sources did not play a role in the investigation. The authors declare that no funds, grants, or other support were received during the preparation of this manuscript.

## CONFLICT OF INTEREST STATEMENT

The authors declare no conflict of interest.

## ETHICS STATEMENT

This article does not contain any studies with human participants or animals performed by any of the authors.
